# Intraspecific Variation in the Rates of Mutations Causing Structural Variation in *Daphnia magna*

**DOI:** 10.1093/gbe/evab241

**Published:** 2021-11-26

**Authors:** Eddie K H Ho, Sarah Schaack

**Affiliations:** Department of Biology, Reed College, Portland, Oregon, USA

**Keywords:** insertion, deletion, duplication, copy number variable, mutation accumulation, *Daphnia*, waterflea, Cladocera

## Abstract

Mutations that cause structural variation are important sources of genetic variation upon which other evolutionary forces can act, however, they are difficult to observe and therefore few direct estimates of their rate and spectrum are available. Understanding mutation rate evolution, however, requires adding to the limited number of species for which direct estimates are available, quantifying levels of intraspecific variation in mutation rates, and assessing whether rate estimates co-vary across types of mutation. Here, we report structural variation-causing mutation rates (svcMRs) for six categories of mutations (short insertions and deletions, long deletions and duplications, and deletions and duplications at copy number variable sites) from nine genotypes of *Daphnia magna* collected from three populations in Finland, Germany, and Israel using a mutation accumulation approach. Based on whole-genome sequence data and validated using simulations, we find svcMRs are high (two orders of magnitude higher than base substitution mutation rates measured in the same lineages), highly variable among populations, and uncorrelated across categories of mutation. Furthermore, to assess the impact of scvMRs on the genome, we calculated rates while adjusting for the lengths of events and ran simulations to determine if the mutations occur in genic regions more or less frequently than expected by chance. Our results pose a challenge to most prevailing theories aimed at explaining the evolution of the mutation rate, underscoring the importance of obtaining additional mutation rate estimates in more genotypes, for more types of mutation, in more species, in order to improve our future understanding of mutation rates, their variation, and their evolution.

Significance

Mutations causing structural variation are important sources of genetic variation, but few direct estimates exist. To understand how mutation rates evolve and expand the current state of knowledge, we must 1) add to the limited number of species for which rates are known, 2) quantify intraspecific variation, and 3) assess whether rate estimates co-vary across types of mutation. We estimate rates of six categories of structural variation-causing mutation in nine genotypes of *Daphnia magna* using a mutation accumulation experiment and short-read whole-genome sequence data. We find rates are high (relative to base substitution mutation rates), highly variable among genotypes, and uncorrelated across categories of mutation, posing a challenge to most prevailing theories aimed at explaining the evolution of mutation rates.

## Introduction

Estimates of mutation rates have focused primarily on base substitution mutation rates, not because they are necessarily representative of the rates for other types of mutations or the highest impact for generating genetic variation, but because they are the easiest to observe (Press et al. 2019). Mutations that result in structural variation (e.g., insertions, deletions, or duplications), however, are abundant and important contributors to the mutational spectrum, even though few direct rate estimates for them exist (Katju and Bergthorsson 2019). Estimating structural variation-causing mutation rates (svcMRs) using short-read whole-genome sequence (WGS) data requires greater depth of coverage and better reference assemblies than what is needed to call single nucleotide changes in order to identify and substantiate breakpoints (Mahmoud et al. 2019). Here, we report mutation rates for six categories of mutation that cause structural variation using high-throughput WGS data generated from mutation accumulation (MA) lines of *Daphnia magna*, a freshwater aquatic microcrustacean. In eukaryotes with sufficiently short generation times, direct estimates of mutation rates using an MA approach represent the gold standard for accurate rate estimation. This is because, as long as they are not lethal or sterilizing, mutations can be observed when selection is minimized by propagating lines via random, single progeny descent.

In addition to expanding the range of rate estimates available, we are interested in measuring levels of intraspecific variation in mutation rates, a parameter that has largely been considered invariant within species heretofore (e.g., Lynch et al. 2016). Determining how mutation rates evolve requires, however, understanding the degree to which mutation rates vary both within and between species, as well as knowing the forces driving and acting on such variation. Intraspecific variation in mutation rates and spectra has only been reported in a few studies (e.g., Adrion et al. 2017), but this is because MA experiments have typically been initiated from only one or a couple genotypes, making an assessment of variation levels impossible (but see Ho et al. 2019, 2020, 2021). In cases where intraspecific variation has been looked for explicitly, for example in yeast, it has been found (e.g., 6-fold differences in base substitution rates based on MA experiments started with five genotypes of *Saccharomyces cerevisiae* [Pankajam et al. 2020]), but data from across taxa is lacking.

Similarly, most papers only report mutation rates for a single type of mutation and a comparison of rate estimates across studies is challenging because sequencing platforms, depth of coverage, and/or methods differ between studies. Given the different mechanisms causing mutations and repairing DNA lesions, one would certainly expect rates to vary among different categories of mutation (Gualberto and Newton 2017). That said, the theories postulating how evolutionary forces shape mutation rate evolution are not specific to one type of mutation or another—that is, lineages with “high” mutation rates for one category (e.g., base substitutions) would be predicted to have relatively high mutation rates in all categories (e.g., insertions or deletions). Indeed, there are many categories of theories aimed at explaining the evolution of mutation rates (ranging from those focusing on life-history characteristics like longevity [e.g., Nabholz et al. 2008] to those arguing population genetic constraints are the major determinant like the Drift Barrier Hypothesis [e.g., Sung et al. 2012]). All of these theories, implicitly, make two predictions: 1) variation between species will be greater than within-species variation and 2) there should be high co-variance among rates for different types of mutations across lineages, even though mechanisms of mutation are diverse, if evolutionary drivers of mutation rate change operate on mutations, regardless of type.

Our previous work in *D. magna* has shown very high levels of intraspecific variation in mutation rates for microsatellites (Ho et al. 2019) and base substitutions (Ho et al. 2020), posing a challenge to the first prediction. Here, we estimate svcMRs in order to test the second prediction, and to add to the small number of direct estimates in eukaryotes available using an MA approach. We initiated MA lines from nine different genotypes of *D. magna* originally collected from three populations (Finland, Germany, and Israel). Specifically, we estimate rates for short insertions and deletions, long deletions and duplications, and deletions and duplications at copy number variable (CNV) sites, in addition to looking at the relationship between structural mutations and their lengths. In the case of gene deletion/duplication rates, there are some estimates from other species indicating rates of svcMRs are high (Katju and Bergthorsson 2013; Konrad et al. 2018; Chain et al. 2019) and highly variable within a species (an order of magnitude difference among rates reported in *Caenorhabditis elegans* [Lipinski et al. 2011; Konrad et al. 2018] and *Daphnia pulex* [Keith et al. 2016; Chain et al. 2019]). Quantifying the tempo at which structural variation is introduced will help uncover the contribution of larger mutations to both deleterious mutation load and the adaptive potential in populations.

## Results

### Mutation Rate Estimates

**Fig. 1.**
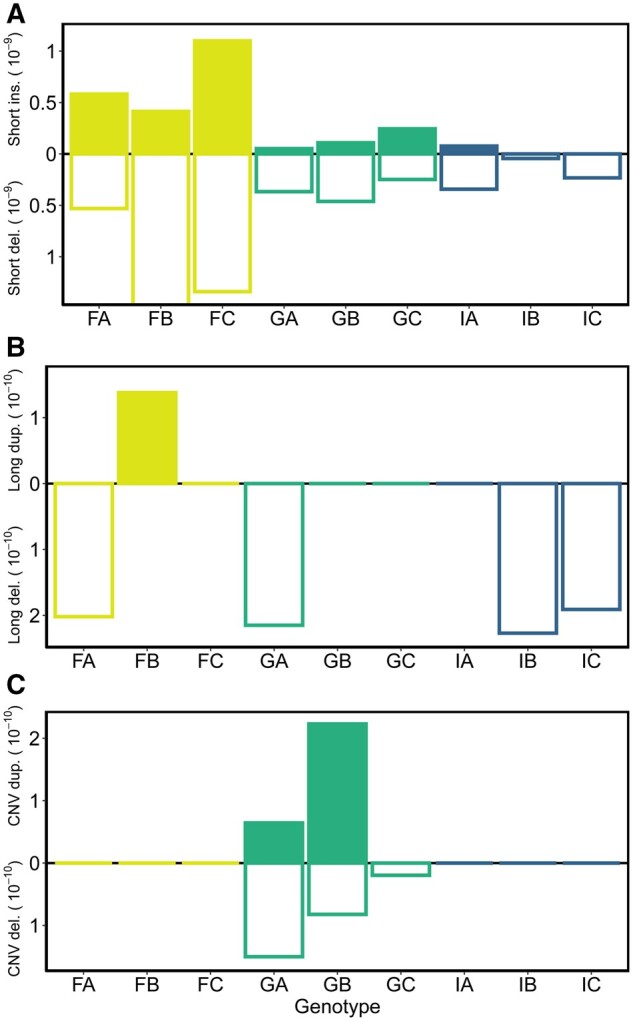
Mutation rates (per bp per generation) for six types of structural variants averaged across MA lines of each genotype. Mutations include (*A*) short indels (<50 bp), (*B*) long deletion and tandem duplications (≥50 bp), and (*C*) CNV deletion and duplications (≥2,000 bp). Yellow, green, and blue represent genotypes that originated from Finland, Germany, and Israel, respectively. The short deletion rate for FB was an order of magnitude larger than other genotypes (supplementary table S4, Supplementary Material online) but we did not extend the scale of the plot for clarity. See supplementary table S4, Supplementary Material online for 95% CIs of the means.

We sequenced the whole genome of MA lines propagated from nine ancestral genotypes originating from three populations (Finland, Germany, and Israel) of *D. magna* (*n* = 9 ancestral lines and *n* = 66 MA lines). The entire experiment consisted of 819 MA generations, with each MA line undergoing an average of 12.4 generations (see supplementary Methods, Supplementary Material online for details). We observed 62 short indels (<50 bp) total (39 deletions and 23 insertions) and used these events to calculate per bp per generation rates of mutation (table 1; see supplementary tables S2 and S3, Supplementary Material online for individual events and line-specific rates). The mean short indel rate was 1.34 × 10^−9^ (95% confidence interval [CI] 6.0–24.1 × 10^−10^) per bp per generation (summing insertion and deletion events), with a ratio of insertion to deletions of 0.59:1. Rates do not differ between insertions and deletions (*t* = 1.65, df = 65, *P* = 0.10; fig. 1*A*, supplementary table S4, Supplementary Material online), nor is there a difference in their average length (table 1 and supplementary table S2, Supplementary Material online; *t* = 0.17, df = 41.1, *P* = 0.87). To examine if the rates differ intraspecifically, we summed the count of indels and fit a binomial generalized linear mixed effect model with population as a fixed effect and MA line (nested within genotype and population) as a random effect (fig. 1). Populations differ in their short indel rates, with Finnish genotypes having higher rates than genotypes from Germany and Israel based on post hoc Tukey’s HSD tests (*χ*^2^ = 34.9, df = 2, *P* < 0.0001; fig. 1*A*, supplementary fig. S1 and table S5A, Supplementary Material online). Longer events (≥50 bp) showed no effect of population using a Kruskal–Wallis test, but were much more rarely observed (six long deletions [ranging from 417 to 5,508 bp] and one 1,697 bp tandem duplication; χ^2^ = 1.72, df = 2, *P* = 0.42; fig. 1*B*, supplementary tables S2 and S3, Supplementary Material online).

**Table 1:** Direct Estimates of svcMRs Based on Short-Read WGS Data from Mutation Accumulation Lines (*n* = 66) Initiated from nine Genotypes of *Daphnia magna* Collected Originally from Finland, Germany, and Israel (rates are in bold, length-adjusted rates are in italics)

				Rates (95% CI) (×10–10) per bp per generation	
		*n*	Mean Length (range)		Difference Among Populations?
Mutation Type			bp	*Length-adjusted (× 10^−8^)*	
Short indels					
(<50 bp)	Insertions	23	3.6 (1–24)	**2.72 (1.4, 4.2)**	
				*0.08 (0.03, 0.1)*	
	Deletions	39	3.8 (1–24)	**10.68 (3.6, 21.5)**	
				*0.3 (0.1, 0.6)*	
	Insertion + Deletions	62	3.7 (1–24)	**13.40 (6.1, 24.1)**	Yes
					*χ* ^2^ = 34.9, df = 2, *P* < 0.0001
					Binomial generalized linear mixed model
Long deletions and duplications					
(≥50 bp)	Tandem duplications	1	1,697 (NA)	**0.06 (0, 0.2)**	
				*1.06 (0, 3.2)*	
	Deletions	6	1,888 (417–5,508)	**0.98 (0.2, 1.9)**	
				*11.81 (3.0, 23.3)*	
	Tandem duplications + Deletions	7	1,861 (417–5,508)	**1.04 (0.3, 2.0)**	No
					*χ* ^2^ = 1.72, df = 2, *P* = 0.42
					Kruskal–Wallis
CNV sites					
	Duplications	42	2,738 (2,000–10,000)	**3.48 (0.3, 8.6)**	
				*95.35 (5.2, 238.0)*	
	Deletions	10	2,400 (2,000–4,000)	**3.05 (0.6, 6.5)**	
				*76.21 (12.6, 162.0)*	
	Duplications + Deletions	52	2,673 (2,000–10,000)	**6.53 (1.6, 12.9)**	Yes
					*χ* ^2^ = 13.44, df = 2, *P* = 0.0012
					Kruskal–Wallis

The number of observed events (*n*), the mean length (bp), the mean rates (with 95% CIs) for six categories of mutation, and the net rates for each category include a test for population effects.

We also detected 52 CNV events (≥2,000 bp in length; 10 deletions and 42 duplications; table 1). CNV events were only detected in MA lines initiated from genotypes from Germany (GA7, GA8, GA10, GB1, GB8, GB10, and GC9) with 75% of CNV events exclusive to a single MA line (39 of the 52 occurred in GB1; fig. 1*C* and supplementary tables S2 and S3, Supplementary Material online). High variance in the number of CNV events across lines has been observed previously in MA experiments (60% in one line for *D. pulex* [Chain et al. 2019]) and 85% in one line for *C. elegans* [Konrad et al. 2018]). Deletions at CNV sites ranged from a 50% to 100% reduction in copy number, but duplications always appeared as a 50% increase in copy number. The mean lengths of CNV deletions and duplications do not differ in our study (*t* = −1.11, df = 27.1, *P* = 0.27) and are similar to those reported for *D. pulex* in Chain et al. (2019; which ranged from 0.6 to 89 kb), although we did not find CNV events as large as those reported in *D. pulex* in Keith et al. (2016; up to 1.4 Mb).

Averaging across all MA lines, the overall rate of CNV mutation was 6.53 × 10^−10^ (95% CI 1.6–12.9 × 10^−10^) per bp per generation (table 1, fig. 1*C*, supplementary table S4, Supplementary Material online), which is very similar to the rate reported in *D. pulex* (6.5 × 10^−10^; Chain et al. 2019). Notably, however, all the CNV mutations occurred in lineages derived from genotypes collected from Germany. Using only the German MA lines, the CNV deletion and duplication rates are 8.4 × 10^−10^ (95% CI 1.8–17.2 × 10^−10^) and 9.6 × 10^−10^ (95% CI 0.7–23 × 10^−10^) per bp per generation, respectively (supplementary fig. S2, Supplementary Material online). These rates are comparable to the rates of short deletion (3.59 × 10^−10^) and short insertion (1.35 × 10^−10^) in the German lines (supplementary fig. S2, Supplementary Material online). A Kruskal–Wallis test on the total CNV rates, unsurprisingly, shows a significant effect of population (*χ*^2^ = 13.44, df = 2, *P* = 0.0012), with German MA lines having a higher CNV mutation rate compared with Finland and Israel (post hoc Dunn test; supplementary table S5B, Supplementary Material online). When looking for correlations among rates across types of mutations, CNV duplication and deletion rates were the only two categories that exhibited even a marginally significant correlation (Pearson’s correlation = 0.62, *t* = 2.13, df = 7, *P* = 0.07), whereas no positive or negative correlations were found among rates for any other mutation types in this study (supplementary table S6, Supplementary Material online). Note that our statistical power to estimate correlations is limited by the low number of genotypes and large number of zeros. However, we believe the lack of correlation is still reflective of the very different patterns observed for how indel and CNV rates vary across genotypes (fig. 1).

### Genomic Impacts

Although mutations generating structural variation are typically reported as the number of events (regardless of size) per base pair per generation, their length can influence their impact. In order to better assess the change introduced into the genome by mutations of various sizes given their frequency, we calculated length-adjusted rates for each category of mutation. Short indels have the lowest rates and larger events, such as CNV mutations, are higher, by up to two orders of magnitude when rates are adjusted for length (table 1; supplementary fig. S3 and table S4, Supplementary Material online). We also calculated the “net change” in genome length due to reductions in length by deletion events and increases in length by duplication events (see Methods section). The net change in base pairs due to deletions, insertions, and duplications ranged from −10 kb (GA7) to +102 kb (GB1) per line. However, for the majority of MA lines (54 out of 66), the net change was <100 bp (supplementary table S2, Supplementary Material online). Overall, net mutation rates were variable, ranging from −2.1 to 3.7 × 10^−5^ bp per bp per generation (supplementary table S7, Supplementary Material online).

In addition to considering length, in many cases we found that mutations at CNV sites overlap genes (7 deletions and 31 duplications out of 52 CNV events total). Thus, averaging across all MA lines, the rates of gene deletion and duplication were 2.81 × 10^−6^ (95% CI 0–7.1 × 10^−6^) and 3.58 × 10^−6^ (95% CI 0–8.6 × 10^−6^) per gene per generation, respectively (table 2). If we only consider cases where CNV changes encompass > 95% of the gene (i.e., “complete” gene deletions/duplications), the rates are lower (1.17 × 10^−6^ [95% CI 0–3.2 × 10^−6^] and 0.27 × 10^−6^ [95% CI 0–0.8 × 10^−6^] per gene per generation, respectively; table 2). For all types of mutations, in order to assess if the frequency of events overlapping genes differs from what one would expect by chance, we simulated an entire set of mutations (matching the number, length, and contig location of the observed set) 1,000 times (see supplementary Methods, Supplementary Material online). The observed number of events (short indels, long deletions and tandem duplications, and CNVs overlapping genes) always falls within the 5th and 95th percentile of the simulated distribution (supplementary fig. S4, Supplementary Material online), suggesting that selection was successfully minimized in the MA experiment.

**Table 2:** Partial and complete^a^ gene duplication and deletion rates per gene per generation for *D. magna* averaged across MA lines from each genotype from Germany (GA, GB, and GC), across all genotypes from Germany, and across the species^b^

Group	Count of CNV Mutations		Rates Including Partial and Complete Genes (95% CI)			Rates for Complete^a^ Genes Only (95% CI)		
	Partially overlap genes	Completely overlap genes	Per gene per generation			Per gene per generation		
			Deletion + Duplication (×10^−8^)	Deletion (×10^−8^)	Duplication (×10^−8^)	Both (×10^−8^)	Deletion (×10^−8^)	Duplication (×10^−8^)
GA	7	2	23.21 (0, 55.47)	16.41 (0, 44.72)	6.79 (0, 20.38)	5.09 (0, 11.32)	2.83 (0, 8.49)	2.26 (0, 6.79)
GB	28	1	29.51 (0, 63.75)	6.79 (0, 20.36)	22.73 (0, 54.6)	6.79 (0, 20.36)	6.79 (0, 20.36)	0
GC	0	0	0	0	0	0	0	0
Germany	35	3	17.57 (4.53, 43.96)	7.73 (0, 18.87)	9.84 (0, 22.73)	3.96 (0, 9.43)	3.21 (0, 8.68)	0.75 (0, 2.26)
*D. magna* ^b^	35	3	6.39 (1.1, 13.26)	2.81 (0, 7.14)	3.58 (0, 8.55)	1.44 (0, 3.50)	1.17 (0, 3.15)	0.27 (0, 0.82)

a A complete gene deletion or duplications requires >95% of the gene being overlapped by a CNV mutation.

b The species-wide estimate is the mean given the total number of MA lines in the experiment from all three populations, Germany, Finland, and Israel, even though no events were observed in MA lines derived from Finland and Israel.

## Discussion

### Variation in svcMRs across Genotypes and Categories of Mutation

Few direct estimates of the rate of structural variation-causing mutations exist, because these events are difficult to observe. Importantly, misassembled or incompletely assembled genomes can make it impossible to detect events in a given lineage, and comparing rates across lineages can be difficult due to biases introduced by reference genomes. In lieu of wet bench validation of large structural variations (where absence of evidence frequently is not evidence of absence), we performed simulations to determine the sensitivity of our methods, but these, too, have limitations and can introduce biases. Despite these challenges, an understanding of mutation rates requires more than just estimates of base substitutions, which may not be representative of rates for other categories of mutation. Here, we report direct estimates for six categories of svcMRs in nine genotypes of *D.magna* collected from three populations across a latitudinal gradient. Theories aimed at explaining how mutation rates evolve typically ignore intraspecific variation and variation in rates among mutation types, because that variation is assumed to be negligible. If variation exists upon those two axes, however, it would be crucial for understanding the evolution of the mutation rate, as a trait. Previously, we showed that *D. magna* has among the highest rates of microsatellite and base substitution mutations reported so far in animals using a mutation accumulation approach (Ho et al. 2019, 2020). These high rates, and the wealth of information on the ecology of *Daphnia*, make this a particularly good system for investigating mutation rate variation (Schaack 2008; Miner et al. 2012).

Our estimates of the rate at which mutations causing structural variation occur in *D. magna* fall within the current known range for eukaryotes (Katju and Bergthorsson 2019; Konrad et al. 2019; Saxena et al. 2019). The short indel mutation rate for *D. magna* (1.34 × 10^−9^ per bp per generation), for example, is near the higher end of the range of rates in other multicellular eukaryotes (between 0.31 and 1.37 × 10^−9^; see supplementary table S8*A* and *B*, Supplementary Material online for metazoan rates from other MA studies and the coefficients of variation for rates calculated for each species). Similarly, the ratio of insertion to deletion events that we observed (0.59:1) and the ratio of rates of base substitutions to indels (6.6:1) in *D. magna* falls within the reported range for eukaryotes (0.17:1–0.65:1 [insertion to deletion] and 3.1–18.1 [base substitution to indel], respectively; supplementary table S8*A* and *B*, Supplementary Material online). It is important to note, our rate estimates represent a conservative lower-bound, as our simulation data revealed that, while the false discovery rate was typically 0 using our pipeline, our false negative rate (FNR) ranged from 4% to 9% in most cases (see supplementary table S9*A* and *B*, Supplementary Material online).

Importantly, we observed significant variation in mutation rates in two dimensions. Within a category of mutation, we observed high levels of intraspecific variation. For example, Finnish MA lines had higher short indel rates than German and Israeli lines (fig. 1*A*, supplementary table S5*A*, Supplementary Material online), the same pattern observed in base substitution mutation rates among these genotypes (Ho et al. 2020). In contrast, CNV mutation rates were highest in German lines and no events were detected in Finnish and Israeli lines (fig. 1*C*, supplementary table S5*B*, Supplementary Material online). These show not only high variation in mutation rates across genotypes and populations, but a remarkable lack of covariation in rates across categories of mutation (supplementary table S6, Supplementary Material online).

To quantify and compare intraspecific variation in mutation rates across species, we calculated the coefficient of variation (CV) of rates for all species where rates have been measured for multiple genotypes using MA (supplementary table S8*B*, Supplementary Material online). Across our nine *D. magna* genotypes, the CV for the indel mutation rate (short insertions + short deletions) is 2.07. For comparison, the CV for indel rates in *D. melanogaster* (Keightley et al. 2009; Schrider et al. 2013; Huang et al. 2016; Sharp and Agrawal 2016) and *C. elegans* (Saxena et al. 2019; Konrad et al. 2019) are 0.60 and 0.87, respectively. The higher CV in rates observed for *D. magna* compared with other species could represent 1) true levels of high intraspecific variation in rates, 2) an artifact of low MA generations elevating the variance of our estimates compared with other studies, or 3) an undersampling of genotypes in the other species (*C. elegans*: two genotypes, *D. melanogaster*: four genotypes, *D. pulex*: three genotypes). However, it is difficult to disentangle these effects given the differences in methodology across studies.

### Genomic Impacts of Mutations Causing Structural Variation

Unlike substitutions, mutations causing structural variation can alter the size of the genome and/or remove or duplicate functional regions. In *D. magna*, length-adjusted rates are higher and more variable among genotypes (table 1), but there is no difference in the net mutation rates (rates incorporating the increase or decrease in bps of each event; ANOVA, *n* = 66, *F*_8,57_ = 0.74, *P* = 0.65, supplementary table S7, Supplementary Material online). In fact, the net mutation rate of *D. magna* is not significantly different from 0 (*t* = 0.12, df = 65, *P* = 0.9; supplementary table S7, Supplementary Material online). This suggests that, in the absence of selection, genome length is stable. We observed a similar result when investigating *D. magna* microsatellite mutations (Ho et al. 2019), in that genotypes that initially had more microsatellite content exhibited a bias toward deletion and genotypes that initially had less microsatellite content exhibited a bias toward insertion.

Although mutations are thought to be deleterious, on average, gene deletions and duplications can also represent an important source of genetic variation and adaptation, playing vital roles in the evolution of organismal complexity (Innan and Kondrashov 2010; Assis and Bachtrog 2013; Katju and Bergthorsson 2013; Panchy et al. 2016). For example, in novel environments, natural populations harbor gene duplicates that are adaptive (Kondrashov 2012) and experimental evolution has shown adaptations involving gene duplications (Andersson and Hughes 2009; Farslow et al. 2015). There is also strong evidence that even gene deletions can be adaptive in natural and laboratory populations (Farslow et al. 2015; Helsen et al. 2020; Monroe et al. 2021).

Notably, the gene deletion and duplication rates of *D. magna* (2.81 × 10^−6^ and 3.58 × 10^−6^ per gene per generation, respectively) are more than two orders of magnitude higher than their base substitution mutation rate (8.9 × 10^−9^ per bp per generation; Ho et al. 2020), a pattern observed in other eukaryotes as well (Katju and Bergthorsson 2013; see supplementary table S8*B*, Supplementary Material online for rates of gene deletion and duplication from other species). Similar to other MA experiments (Lipinski et al. 2011; Schrider et al. 2013), only a few gene deletion/duplication events in our study encompass an entire gene (3 of 38), but even partial events can confer adaptive loss of function or lead to sub- or neofunctionalization (Innan and Kondrashov 2010). Intraspecific variation in gene deletion and duplication rates in *D. magna* (CV = 2.00) are greater than that seen in *C. elegans* (Lipinski et al. 2011; Konrad et al. 2019) and *D. pulex* (Keith et al. 2016; Chain et al. 2019), where they are 1.37 and 1.03, respectively.

Our estimates of svcMRs in *D. magna* reveal 1) high levels of intraspecific variation and 2) differences in rates among categories of mutation that do not co-vary across genotypes. In addition to challenging most theories aimed at explaining the evolution of the mutation rate, our data add to the small list of taxa for which direct estimates are available. Ultimately, variation among populations in the rate and type of mutations can lead to distinct evolutionary trajectories and diversification. Theoretical work shows that the rate of fixation for adaptive mutations is proportional to the mutation rate (Neher et al. 2010). Similarly, different mutation loads resulting from deleterious mutations can alter the risk of extinction (Lynch et al. 1993), especially in asexually reproducing organisms. In *Daphnia*, a genus where many species reproduce via cyclical parthenogenesis, the frequency of sexual reproduction can vary, influencing the efficacy of selection (Gerber et al. 2018) and leading to the evolution of variable recombination rates (Kondrashov 1988; Otto 2009). Our findings underscore the importance of measuring mutation rates for multiple types of mutations, from multiple genotypes, in more species. Ultimately, understanding factors affecting the evolution of the mutation rate depend on a comprehensive set of estimates for this critical trait.

## Materials and Methods

For detailed methods see supplementary Methods, Supplementary Material online. In brief, we performed MA experiments using nine starting genotypes of *D. magna* from three populations (Finland, Germany, and Israel; supplementary table S1, Supplementary Material online). Lines were propagated using single-progeny descent (one individual offspring transferred each generation) in order to minimize selection. Tissue from all MA lines generated (*n* = 66 total) and tissue from ancestors (“starting genotype”) was frozen and used for whole-genome sequencing. DNA libraries were prepared and sequenced using the Illumina platform to produce ∼50× coverage genome-wide for each sample. Sequence data were assembled and annotated in order to identify genic regions. Reads from each MA line were mapped to the genome assembly of their respective starting genotype using BWA (Li and Durbin 2009) and with SpeedSeq (Chiang et al. 2015) to output discordant and split reads. Only sites that met stringent quality filters were included in the downstream analysis, which reduced the region of the genome in which it was possible to call variants to between 53 and 71 Mb across the nine genotypes (supplementary table S1, Supplementary Material online).

Several programs were used to call short indels (<50 bp), long deletions and tandem duplications (≥50 bp), and CNV changes (≥2,000 bp) in order to calculate mutation rates for each category of mutation. We used simulations in order to estimate false discovery and FNRs (see supplementary Methods, Supplementary Material online for complete details) for different categories of events. In addition, we plotted events by their length (supplementary fig. S5, Supplementary Material online) and calculated length-adjusted mutation rates, in order to incorporate the size of the mutation into the estimate. Mutation rate for each MA line was calculated as $u_{i}=\;x_{i}/(2\;g\;n)$, where *x_i_* represents the number of events for structural variant type *i*, *g* represents the number of MA generations, and *n* represents the number of callable sites. The length-adjusted mutation rate for each MA line was calculated as

$$v_{i}\;=\;\sum_{j}l_{i,j}/(2\;g\;n),$$

where *l_i, j_* represents the length of the *j*th event for SV type *i*. To examine the overall effect of SVs on genome length, we calculated the total-length and the net-length rate for each MA line as,

$$\sum_{i}v_{i}\;\mathrm{and}\sum_{i}c_{i}v_{i}$$

respectively, where *c_i_* equals +1 for insertions/duplications and −1 for deletions. In addition, we calculated the genic effects of structural variants by examining CNV events that overlapped genes. For each MA line, the per gene rate was calculated as $w_{i}\;=\;y_{i}/(2\;g\;m)$, where *y_i_* represents the number of genes overlapped by structural variant type *i*, and *m* represents the total number of genes in each assembly. We also examined whether structural variants overlapped genes more or less often than expected by chance, by simulating a set of mutations for each MA line by re-sampling (see supplementary Methods, Supplementary Material online for full details). Lastly, we compared intraspecific variation in mutation rates by calculating the CV for rates across our *D. magna* genotypes, as well as for species where rates were reported for more than one genotype.

## Supplementary Material

Supplementary data are available at *Genome Biology and Evolution* online.

### Data Availability

All WGS data have been deposited at NCBI (PRJNA658680), and all code are available online (https://github.com/EddieKHHo/simMutAccumSV, last accessed September 2021).

## Supplementary Material

evab241_Supplementary_Data

## References

[evab241-B1] Adrion JR , Song MJ, Schrider DR, Hahn MW, Schaack S. 2017. Genome-wide estimates of transposable element insertion and deletion rates in *Drosophila melanogaster*. Genome Biol Evol. 9(5):1329–1340. 28338986 10.1093/gbe/evx050 PMC5447328

[evab241-B2] Andersson DI , Hughes D. 2009. Gene amplification and adaptive evolution in bacteria. Annu Rev Genet. 43:167–195. 19686082 10.1146/annurev-genet-102108-134805

[evab241-B3] Assis R , Bachtrog D. 2013. Neofunctionalization of young duplicate genes in *Drosophila*. Proc Natl Acad Sci USA. 110(43):17409–17414. 24101476 10.1073/pnas.1313759110 PMC3808614

[evab241-B4] Chain FJJ , Flynn JM, Bull JK, Cristescu ME. 2019. Accelerated rates of large-scale mutations in the presence of copper and nickel. Genome Res. 29(1):64–73. 30487211 10.1101/gr.234724.118 PMC6314161

[evab241-B5] Chiang C , et al 2015. SpeedSeq: ultra-fast personal genome analysis and interpretation. Nat Methods. 12(10):966–968. 26258291 10.1038/nmeth.3505 PMC4589466

[evab241-B6] Farslow JC , et al 2015. Rapid increase in frequency of gene copy-number variants during experimental evolution in *Caenorhabditis elegans*. BMC Genomics. 16:1044. 26645535 10.1186/s12864-015-2253-2 PMC4673709

[evab241-B7] Gerber N , Kokko H, Ebert D, Booksmythe I. 2018. *Daphnia* invest in sexual reproduction when its relative costs are reduced. Proc R Soc B. 285(1871):20172176. 10.1098/rspb.2017.2176 PMC5805931 29343596

[evab241-B8] Gualberto JM , Newton KJ. 2017. Plant mitochondrial genomes: dynamics and mechanisms of mutation. Annu Rev Plant Biol. 68:225–252. 28226235 10.1146/annurev-arplant-043015-112232

[evab241-B9] Helsen J , et al 2020. Gene loss predictably drives evolutionary adaptation. Mol Biol Evol. 37(10):2989–3002. 32658971 10.1093/molbev/msaa172 PMC7530610

[evab241-B10] Ho EKH , et al 2021. Engines of change: transposable element mutation rates are high and variable within*Daphnia magna*. PLoS Genet. 17(11):e1009827. 10.1371/journal.pgen.1009827 PMC8594854 34723969

[evab241-B11] Ho EKH , et al 2019. Intraspecific variation in microsatellite mutation profiles in *Daphnia magna*. Mol Biol Evol. 36(9):1942–1954. 31077327 10.1093/molbev/msz118 PMC6934441

[evab241-B12] Ho EKH , et al 2020. High and highly variable spontaneous mutation rates in *Daphnia*. Mol Biol Evol. 37(11):3258–3266. 32520985 10.1093/molbev/msaa142 PMC7820357

[evab241-B13] Huang W , et al 2016. Spontaneous mutations and the origin and maintenance of quantitative genetic variation. eLife 5:e14625. 27213517 10.7554/eLife.14625 PMC4929002

[evab241-B14] Innan H , Kondrashov F. 2010. The evolution of gene duplications: classifying and distinguishing between models. Nat Rev Genet. 11(2):97–108. 20051986 10.1038/nrg2689

[evab241-B15] Katju V , Bergthorsson U. 2013. Copy-number changes in evolution: rates, fitness effects and adaptive significance. Front Genet. 4:273. 24368910 10.3389/fgene.2013.00273 PMC3857721

[evab241-B16] Katju V , Bergthorsson U. 2019. Old trade, new tricks: insights into the spontaneous mutation process from the partnering of classical mutation accumulation experiments with high-throughput genomic approaches. Genome Biol Evol. 11(1):136–165. 30476040 10.1093/gbe/evy252 PMC6330053

[evab241-B17] Keith N , et al 2016. High mutational rates of large-scale duplication and deletion in *Daphnia pulex*. Genome Res. 26(1):60–69. 26518480 10.1101/gr.191338.115 PMC4691751

[evab241-B18] Keightley PD , et al 2009. Analysis of the genome sequences of three *Drosophila melanogaster* spontaneous mutation accumulation lines. Genome Res. 19(7):1195–1201. 19439516 10.1101/gr.091231.109 PMC2704435

[evab241-B19] Kondrashov AS. 1988. Deleterious mutations and the evolution of sexual reproduction. Nature 336(6198):435–440. 3057385 10.1038/336435a0

[evab241-B20] Kondrashov FA. 2012. Gene duplication as a mechanism of genomic adaptation to a changing environment. Proc Biol Sci. 279(1749):5048–5057. 22977152 10.1098/rspb.2012.1108 PMC3497230

[evab241-B21] Konrad A , Brady MJ, Bergthorsson U, Katju V. 2019. Mutational landscape of spontaneous base substitutions and small indels in experimental *Caenorhabditis elegans* populations of differing size. Genetics 212(3):837–854. 31110155 10.1534/genetics.119.302054 PMC6614903

[evab241-B22] Konrad A , et al 2018. Mutational and transcriptional landscape of spontaneous gene duplications and deletions in *Caenorhabditis elegans*. Proc Natl Acad Sci USA. 115(28):7386–7391. 29941601 10.1073/pnas.1801930115 PMC6048555

[evab241-B23] Li H , Durbin R. 2009. Fast and accurate short read alignment with Burrows-Wheeler transform. Bioinformatics 25(14):1754–1760. 19451168 10.1093/bioinformatics/btp324 PMC2705234

[evab241-B24] Lipinski KJ , et al 2011. High spontaneous rate of gene duplication in *Caenorhabditis elegans*. Curr Biol. 21(4):306–310. 21295484 10.1016/j.cub.2011.01.026 PMC3056611

[evab241-B25] Lynch M , Burger M, Butcher D, Gabriel W. 1993. The mutational meltdown in asexual populations. J Hered. 84(5):339–344. 8409355 10.1093/oxfordjournals.jhered.a111354

[evab241-B26] Lynch M , et al 2016. Genetic drift, selection and the evolution of the mutation rate. Nat Rev Genet. 17(11):704–714. 27739533 10.1038/nrg.2016.104

[evab241-B9525662] Mahmoud M , et al 2019. Structural variant calling: the long and the short of it. Genome Biol. 20(1):246. 31747936 10.1186/s13059-019-1828-7 PMC6868818

[evab241-B2349371] Miner BE , De Meester L, Pfrender ME, Lampert W, Hairston NG. 2012. Linking genes to communities and ecosystems: *Daphnia* as an ecogenomic model. Proc Biol Sci. 279(1735):1873–1882. 22298849 10.1098/rspb.2011.2404 PMC3311900

[evab241-B27] Monroe JG , McKay JK, Weigel D, Flood PJ. 2021. The population genomics of adaptive loss of function. Heredity (Edinb) 126(3):383–395. 33574599 10.1038/s41437-021-00403-2 PMC7878030

[evab241-B28] Neher RA , Shraiman BI, Fisher DS. 2010. Rate of adaptation in large sexual populations. Genetics 184(2):467–481. 19948891 10.1534/genetics.109.109009 PMC2828726

[evab241-B29] Nabholz B , Glemin S, Galtier N. 2008. Strong variations of mitochondrial mutation rate across mammals—the longevity hypothesis. Mol Biol Evol. 25(1):120–130. 17998254 10.1093/molbev/msm248

[evab241-B30] Otto SP. 2009. The evolutionary enigma of sex. Am Nat. 174(S1):S1–S14. 19441962 10.1086/599084

[evab241-B31] Panchy N , Lehti-Shiu M, Shiu SH. 2016. Evolution of gene duplication in plants. Plant Physiol. 171(4):2294–2316. 27288366 10.1104/pp.16.00523 PMC4972278

[evab241-B40189918] Pankajam AV , Dash S, Saifudeen A, Dutta A, Nishant KT. 2020. Loss of heterozygosity and base mutation rates vary among *Saccharomyces* cerevisiae hybrid strains. G3 (Bethesda) 10(9):3309–3319. 32727920 10.1534/g3.120.401551 PMC7466981

[evab241-B32] Press MO , Hall AN, Morton EA, Queitsch C. 2019. Substitutions are boring: some arguments about parallel mutations and high mutation rates. Trends Genet. 35(4):253–264. 30797597 10.1016/j.tig.2019.01.002 PMC6435258

[evab241-B33] Saxena AS , Salomon MP, Matsuba C, Yeh SD, Baer CF. 2019. Evolution of the mutational process under relaxed selection in *Caenorhabditis elegans*. Mol Biol Evol. 36(2):239–251. 30445510 10.1093/molbev/msy213 PMC6367967

[evab241-B37] Schaack S . 2008. Daphnia comes of age: an ecological model in the genomic era. Mol Ecol. 17(7):1634–1635. 18266625 10.1111/j.1365-294X.2008.03698.x PMC2740638

[evab241-B34] Schrider DR , Houle D, Lynch M, Hahn MW. 2013. Rates and genomic consequences of spontaneous mutational events in *Drosophila melanogaster*. Genetics 194(4):937–954. 23733788 10.1534/genetics.113.151670 PMC3730921

[evab241-B35] Sharp NP , Agrawal AF. 2016. Low genetic quality alters key dimensions of the mutational spectrum. PLoS Biol. 14(3):e1002419. 27015430 10.1371/journal.pbio.1002419 PMC4807879

[evab241-B36] Sung W , Ackerman MS, Miller SF, Doak TG, Lynch M. 2012. Drift-barrier hypothesis and mutation-rate evolution. Proc Natl Acad Sci USA. 109(45):18488–18492. 23077252 10.1073/pnas.1216223109 PMC3494944

