# Supplementary material for: Intraspecific Variation in the Rates of Mutations Causing Structural Variation in Daphnia magna
Source: Genome Biol Evol. 2021 Nov 26;13(12):evab241. doi: 10.1093/gbe/evab241 (PMC8691059; doi:10.1093/gbe/evab241)
Supplement: evab241_Supplementary_Data [file evab241_supplementary_data.zip › 101221_SuppMaterials_Figures.pdf]

SUPPLEMENTAL MATERIALS

Supplemental Methods

Study System

The *D. magna* genotypes used in this experiment were selected from populations along a latitudinal gradient in order to sample individuals originating from a broad range of environments (provided by D. Ebert). Different maximum and mean temperatures and photoperiods, both of which can also result in fluctuating habitat sizes (Yampolsky et al. 2014), are represented by the three populations (Finland, Germany, and Israel; described in Ho, Bellis, et al. bioarxiv/in revision for PLoS Genetics.).

Experimental Design

Three genotypes from each of three populations (Finland, Germany, and Israel) were used to initiate laboratory stocks. From these lab stocks, starting controls (SCs) were selected (immediate descendants of which were frozen and sequenced) for each of the 9 genotypes. From the SCs, mutation accumulation (MA) lines (n = 5-12 per genotype; total of 66) and large population controls (extant controls [ECs]; n = 2 per genotype; total of 18) were initiated and propagated in parallel. Tissue from each line (MAs and ECs) was frozen regularly during the mutation accumulation period; the average number of generations across MA lines was 12 and the experiment ran for approximately 30 months in total (Table S1).

The MA and EC lines from each genotype were maintained as single individuals or large populations in 250 mL beakers containing 175-200 mL or 3.5 L jars containing 3 L of Aachener Daphnien Medium (ADaM; Klüttgen et al. 1994), respectively. All lines were maintained under a constant photoperiod (16L:8D) and temperature (18° C), and fed the unicellular green alga *Scenedesmus obliquus* (2-3 times per week *ad libitum*). While selection is permitted to act in the large population ECs, the single-progeny descent used to propagate the MA lines maximizes chance and minimizes selection, and thus allows for the accumulation of mutations. The experimental protocols used here have been described previously (Ho et al. 2019, 2020). Importantly, if MA lines went extinct,

tissue from the most recent regular tissue collection was used for DNA extraction and sequencing.

DNA Extraction and Sequencing

At the end of the mutation accumulation period, the 9 SCs, 66 MA lines, and 18 ECs were sequenced. Five asexually-produced clonal individuals from each SC line, all derived MA lines, and the extant control lines were flash frozen for DNA. Libraries were used to generate approximately 50x coverage genome-wide for each sample.

Reference-guided genome assemblies

To reduce reference bias, we built reference-guided assemblies for each of the 9 genotypes (using the WGS from each SC). Reads from each sample were processed by trimming adaptor sequences (k=23, ktrim=r, mink=4, hdist=1, tpe, tbo), merging overlapping pairs (vstrict=t), and quality filtering (qtrim = rl, trimq=20, minlen=50) with BBTools (Bushnell et al. 2017). First pass reference-guided *de novo* assemblies were performed with SPAdes (using the *trusted-contigs* option; Bankevich et al. 2012). The *D. magna* reference used to guide the assembly was provided by Peter Fields and Dieter Ebert (*personal communication*). To remove haplotigs potentially derived from assembling heterozygous regions into alternate alleles, we collapsed the filtered assemblies with *redundans* by implementing information from paired-end reads, merged reads, and the *D. magna* reference genome for scaffolding (Pryszcz and Gabaldón 2016). We then mapped the processed reads of each SC onto the collapsed assemblies with BWA-MEM v0.7.17 (Li and Durbin 2010). Contigs were removed if they possessed an average depth of coverage <5 or were shorter than 5 kb. See Table S10 for genome assembly statistics.

Genome annotation

Annotation was performed separately for each of the nine assemblies. Gene model prediction was conducted by the MAKER pipeline (Cantarel et al. 2008), integrating *ab-initio* prediction with transcripts and protein datasets. For MAKER, we initially constructed gene models by aligning *Daphnia* transcriptome datasets (wfleabase: *D.*

galeata, D. magna, D. pulex) and *Crustacea* proteome datasets (Uniprot database: *Penaeus vannamei, Armadillidium nasatum, Portunus trituberculatus, Tigriopus californicus, Amphibalanus amphitrite, Armadillidium vulgare, D. pulex, D. magna*) to the repeat-masked assembly. Repeats were identified by RepeatModeler (Smit & Hubley 2008) and RepeatMasker (Smit et al. 2013) (described below). We then filtered for gene models with AED values <= 0.25 and used these as input for the *ab-initio* gene predictors AUGUSTUS (Stanke et al. 2008) and SNAP (Korf 2004). The next round of MAKER then incorporated *ab-initio* prediction with the alignment evidence in the first round. We repeated this process for a total of three rounds and identified 15228 to 22072 genes across the nine assemblies.

We further processed these gene annotations to identify single-copy genes. If genes overlapped one another, we only kept the gene with the lower AED value. We then performed a BLAST (Altschul et al. 1990) search of each gene set to itself. If any gene had at least 50% of its length matched with another gene and an e-value of 10^{-10}, it was considered a multi-copy gene and discarded. After these filters, we recovered 7720 to 11340 single copy genes across the nine assemblies (Table S1).

Read mapping

Reads for each sample were mapped to the genome assembly of their respective genotype using BWA (Li and Durbin 2009) and with SpeedSeq (Chiang et al. 2015) to output discordant and split reads. PCR and optical duplicates were removed with Picard MarkDuplicates (http://broadinstitute.github.io/picard).

Removing repetitive regions and counting callable sites

A custom *D. magna* TE consensus library was created from a concatenated file of the assemblies from the 9 genotypes' SCs using RepeatModeler v1.0.11 (Smit & Hubley 2008). Each assembly was then masked with our custom TE consensus library, using the slow search setting of RepeatMasker v4.1.0 (Smit et al. 2013). This filter removed 20 to 24 Mb of the genome from analysis.

We also detected many low-quality regions that contained unusually high amounts of in-phase heterozygous variants. These regions are most likely collapsed duplications in the reference genome. To remove these regions, we utilized GATK *HaplotypeCaller* (McKenna et al. 2010) to genotype each line separately, *GenotypeGVCFs* to perform joint genotyping, and *SelectVariants* to output all biallelic SNPs. For each genotype, we then constructed a consensus sequence across all lines where a site is designated as heterozygous in the consensus if more than two lines support heterozygosity at that site. We then performed a sliding window analysis on heterozygosity of the consensus sequence using 500 bp windows with 100 bp steps. After trying multiple filtering criteria, we decided to assign all 500 bp windows with 7 or more heterozygous sites as potentially problematic. Increasing the stringency of this filter will remove well assembled regions that happen to have higher than average heterozygosity. This filter removed 7 to 15 Mb of the genome from analysis.

Finally, for each genotype we designated a site in the genome as callable if: i) it does not overlap any of the repetitive regions identified above and ii) at most two lines fail to have 20x coverage at the site. This resulted in 53 to 71 Mb of the genome being callable across the 9 genotypes (Table S1).

Calling short indels (< 50 bp)

For each genotype, we utilized GATK *HaplotypeCaller* (McKenna et al. 2010) to genotype each line separately, *GenotypeGVCFs* to perform joint genotyping, and *SelectVariants* to output biallelic indels. For each line, we required that all sites have a depth of coverage between 0.5x and 1.5x of the median coverage calculated across all lines of the genotype. If the site was heterozygous, we additionally required that the second largest phred-likelihood score be ≥ 30.

For each genotype, a novel indel mutation was called if one MA line had a new insertion or deletion allele less than 50 bp long and all other MA lines possessed the same genotype as the ancestral line. In addition, we examined the proportion of the indel supporting reads at “confidently” heterozygous indel sites (i.e., sites that are heterozygous for an indel in SC and all MA lines). We compared the proportion of reads

supporting the new indel to this distribution (based on "confidently" heterozygous indels) and if the proportion fell between the 5th and 95th percentile, the new indel was kept. Finally, we also genotyped lines using *Samtools mpileup* (Li et al. 2009). The new indel was discarded if: i) there were any reads in the mutant MA line that supported an alternated indel sequence, or ii) any reads in the SC or unmutated MA lines supported an indel or base substitution event. Finally, each new indel was manually inspected with IGV and were discarded if: i) reads supported an indel at the focal site, but showed polymorphism at one or more nearby sites, or ii) there were other nearby indels, or iii) indels overlapped discordant or split reads.

Calling long deletions and tandem duplications (≥ 50 bp)

For each genotype, we utilized LUMPY (Layer et al. 2014) to genotype each line separately and SVTyper (Chiang et al. 2015) for joint genotyping. We only kept sites with ≥ 3 discordant or ≥ 3 split reads supporting a deletion or tandem duplication. We then utilized the fraction of reads supporting the deletion/tandem duplication allele (i.e., allele balance; AB) to determine if a line was homozygous for the reference (0/0), heterozygous (0/1) or homozygous for the deletion/tandem duplication allele (1/1).

For deletions, we utilized a slightly more stringent range of AB than that used by LUMPY by default. The line was designated as 0/0, 0/1, 1/1 if the value of AB was within these ranges [0, 0.087], (0.13, 0.62], (0.78, 1], respectively. [0, 0.087] represents the 0th to 97.5th percentile of AB for 0/0 identified by LUMPY, (0.13, 0.62] represents the 2.5th and 97.5th percentile of AB for 0/1 identified by LUMPY, and (0.78, 1] represents the 2.5th and 100th percentile of AB for 1/1 identified by LUMPY.

For tandem duplications, we noticed that LUMPY had difficulty differentiating 0/1 from 1/1 genotypes (based on simulations below), which would impact our ability to detect novel heterozygous and loss of heterozygous mutations. To correct for this, we simulated the MA experiment and processed the data using our pipeline to determine the ranges of AB that best recovered mutations. For each simulation, we generated 11

unique diploid individuals by simulation SNPs onto the largest contig of the FASC assembly (6.8 Mb) using pIRS v1.1.1. (Hu et al. 2012). The 11 individuals represented one ancestral line and 10 descendant MA+EC lines. We then utilized Svsim (https://github.com/GregoryFaust/SVsim) to insert 5 of each type of mutational event: 0/0 -> 0/1 novel tandem duplication, 0/1 -> 0/0 loss of heterozygosity, and 0/1 -> 1/1 loss of heterozygosity. For each mutation, we randomly picked a location on the assembly to duplicate and ensured none overlapped each other. To simulate a 0/0 -> 0/1 novel tandem duplication, we inserted a heterozygous tandem duplication site (i.e., on one homolog of the assembly) into one of the descendent lines. To simulate 0/1 -> 0/0 loss of heterozygosity event, we inserted a heterozygous tandem duplication site on the ancestral and all non-focal descendant lines and did not change the focal descendent line. To simulate 0/1 -> 1/1 loss of heterozygosity event, we inserted a heterozygous tandem duplication site on the ancestral and all non-focal descendant lines and inserted a homozygous tandem duplication site on the focal MA line. Finally, we independently simulated pair-end reads for each of the 11 individuals with an average coverage of 50x using pIRS (Hu et al. 2012) and processed it as described above. This was repeated 20 times each for tandem duplications with lengths {50, 100, 200, 300, 400, 500, 600, 700, 800, 900, 1000, 2000, 3000, 4000, 5000, 6000, 7000, 8000, 9000, 10000} bp, which totals to 6000 of mutational events.

We are only interested in the threshold value of AB that differentiates 0/1 from 1/1 genotypes, which was 0.26 in LUMPY by default. With the threshold value at 0.26, we only recovered 2535 of the 6000 mutations (42.3%) using our pipeline. After trying out a range of values, we found that an AB threshold of 0.32 maximized the number of mutations recovered (4450 of 6000 or 74.2%; Table S11). In summary, for tandem duplications, we designated a line as 0/0, 0/1, 1/1 if the value of AB was within these ranges [0, 0.056], (0.079, 0.32], (0.32, 1], respectively. [0, 0.056] represents the 0th to 97.5^{th} percentile of AB for 0/0 identified by LUMPY. 0.079 represents the 2.5^{th} percentile of AB for 0/1 identified by LUMPY. 0.32 represents the optimal threshold value of AB for differentiating 0/1 from 1/1 based on our simulations.

After assigning genotypes to each line based on the mutation type (deletion/tandem duplication) and their value of AB, we called a novel mutation if one MA line was heterozygous for a deletion/tandem duplication allele that was ≥ 50 bp long while the SC and all other MA lines was homozygous for the reference genotype. We additionally required that the mutant MA line has at least 3 discordant or 3 split reads supporting the novel mutation. Each novel mutation was manually inspected with IGV and were discarded if it overlapped discordant or split reads that supported another type of mutation in any of the lines.

Calling CNVs (≥ 2000 bp)

We utilized cn.MOPS (Klambauer et al. 2012) and CNV-seq (Xie and Tammi 2009) to detect CNVs using a read-depth approach. CNV-seq detects CNV by performing pairwise comparisons, while cn.MOPS can compare more than two samples at once which lowers the false discovery rates (Klambauer et al. 2012).

For each genotype, we ran cn.MOPS with 500 bp sliding windows to detect CNVs across all SC, MA and EC lines. We called a novel CNV mutation if: i) CNV is ≥ 2000 bp long, ii) CNV was at least 2000 bp away from ends of contig, iii) CNV and flanking 4000 bp region contains no more than 10% gaps, iv) CNV is unique to one MA line and does not overlap CNVs in any other line within 4000 bp upstream or downstream. To further reduce the chance of a false positive detection, we required the CNV mutations identified by cn.MOPS corroborated with results from CNV-seq. We ran CNV-seq with 500 bp sliding windows for all possible pairwise combinations of SC, MA and EC lines. CNVs were called if all windows within a 2000 bp region had a $\log_2$ depth of coverage difference above 0.44 or below -0.62, which requires a coverage ratio of > 1.35 or < 0.65, respectively. For each novel CNV mutation detected in cn.MOPS, we required that the mutant MA line possessed an overlapping CNV of the same type (deletion/duplication) in all pairwise comparisons from CNV-seq.

Each novel CNV region was manually inspected with IGV and were discard if: i) it overlapped discordant or split reads that supports another type of mutation, or ii) it was a CNV duplication that overlapped with SNPs that experienced a loss of heterozygosity

(LOH) mutation. The latter condition was imposed because we cannot reconcile why a duplication event will be correlated with a LOH event. We detected 25 CNV duplications in GA7, GA10, GB8, and GB10 that overlapped SNPs that lost heterozygosity.

As shown in the results, we only detected CNV mutations in the genotypes from Germany. To show that the CNVs mutations detected did not simply occur in regions unique to the German assemblies, we performed BLAST searches (Altschul et al. 1990) of each CNV region to each of the 9 SC assemblies. For each search, we kept the match with the longest length and an e-value of less than 10^{-10}. We also reported the mean depth of coverage for the region that matched with the CNV in each assembly. Mean depth of coverage was based on the coverage when mapping the reads from the SC lines on to their respective assemblies (Table S12), and the results indicate there is no reason to suggest that regions prone to CNV events were only sampled in one genotype or that such regions were not sufficiently sequenced to detect CNV sites in other genotypes.

Gene overlap

To examine whether structural variants overlapped genes more or less often than expected by chance, we simulated a set of mutations for each MA line by re-sampling. For each MA line, we sampled the same number of structural variants as observed, matching their lengths and the contigs they occurred on and ensured that they did not overlap. For CNVs, we additionally required that the resampled region possessed a mean depth of coverage within 0.9x and1.1x of the original. After the mutations were sampled, we determined the number of genes that they overlapped. This process was repeated 1000 times to obtain a distribution for the number of genes overlapped by randomly sampled mutations.

Mutation rate estimation

We calculated two different mutation rates for short indels, long deletion/tandem duplications, and CNVs. The first type is the standard rate calculation based on the number of events, while the second type weighs the rate by the length of the structural

variant to account for its effect on the genome length. Mutation rate for each MA line was calculated as $u_i = x_i / (2\ g\ n)$, where x_i represents the number of events for structural variant type *i*, *g* represents the number of MA generations, and *n* represents the number of callable sites. The length-adjusted mutation rate for each MA line was calculated as

$$v_i = \sum_j l_{i,j} / (2\ g\ n)$$

, where $l_{i,j}$ represents the length of the jth event for SV type *i*. To examine the overall effect of SVs on genome length, we calculated the total-length and the net-length rate for each MA line as

$$\sum_i v_i \text{ and } \sum_i c_i\, v_i$$

respectively, where c_i equals +1 for insertions/duplications and -1 for deletions. Lastly, we calculated the genic effects of structural variants by examining CNVs events that overlapped genes. For each MA line, the per gene rate was calculated as $w_i = y_i / (2\ g\ m)$, where y_i represents the number of genes overlapped by structural variant type *i*, and *m* represents the total number of genes in the assembly. We compared intraspecific variation in mutation rates by calculating the coefficient of variation (CV) for rates across our *D. magna* genotypes as well as for species where rates were measured for more than one genotype (Table S8A, 8B). To examine if the rates of short indels differ intraspecifically, we summed the count of short indels and fit a binomial generalized linear mixed effect model with population as a fixed effect and MA line (nested within genotype and population) as a random effect using this formula: glmer(c(Mutation count, Callable sites) ~ Population + (1 | Population : Genotype : Line)). This analysis utilized the lme4 package from R (R Core Team 2018).

Estimating false discovery and false negative rates

The false discovery rate (FDR) represents the proportion of detected mutations that are false positives, while the false negative rate (FNR) represents the proportion of mutations that were undetected. To estimate FDR and FNR, we simulated the MA experiment and processed the data using the pipelines outlined above. For each simulation, we generated 11 unique diploid individuals by simulating SNPs onto the largest contig of the FASC assembly (6.8 Mb) using pIRS v1.1.1 (He et al. 2012). The 11 individuals represented one ancestral line and 10 descendent (MA + EC) lines. We then utilized Svsim (https://github.com/GregoryFaust/SVsim) to insert 5 novel deletions, novel tandem duplications, or novel non-tandem duplications into one of the descendant lines. To simulate a novel SV mutation, we inserted a heterozygous SV site (i.e., on one homolog of the assembly) into one of the descendent lines. We also insert 10 heterozygous sites that are shared amongst the ancestral and all descendant lines. Finally, we independently simulated pair-end reads for each of the 11 individuals with an average coverage of 50x using pIRS (Hu et al. 2012). The Python for the simulations can be found at https://github.com/EddieKHHo/simMutAccumSV.

To estimate FDR and FNR for the pipeline that utilized the LUMPY software (Layer et al. 2014), the simulations were repeated 20 times for deletions and tandem duplications with lengths {50, 100, 200, 300, 400, 500, 600, 700, 800, 900, 1000, 2000, 3000, 4000, 5000, 6000, 7000, 8000, 9000, 10000} bp for a total of 2000 deletions and 2000 tandem duplications (Table S9A). To estimate FDR and FNR for the pipeline that detected CNV mutations using cn.MOPS (Klambauer et al. 2012) and CNV-seq (Xie and Tammi 2009), the simulations were repeated 20 times for deletions and non-tandem duplications with lengths {2000, 2500, 3000, 3500, 4000, 4500, 5000, 5500, 6000, 6500, 7000, 7500, 8000, 8500, 9000, 9500, 10000, 10500} bp for a total of 1800 deletions and 1800 non-tandem duplications. We additionally simulated novel homozygous deletions for the CNV pipeline (i.e. deletions on both homologs of the assembly). This was repeated 40 times for deletions with lengths {2000, 2500, 3000, 3500, 4000, 4500, 5000, 5500, 6000, 6500, 7000, 7500, 8000, 8500, 9000, 9500, 10000, 10500} bp for a total of 3600 complete deletions (Table S9B).

FDR for a mutation type was estimated as FP / (FP + TP), where TP is the number of simulated mutations that were detected and FP is the number of detected SV mutations that did not correspond to a location of a simulated mutation. FNR for a mutation type was estimated as FN / (FN + TP), where FN is the number of simulated mutations that were not identified (Table S9A, B). For our LUMPY pipeline, we found that the FDR was very low (0 and 0.004 for deletions for tandem duplications, respectively) and FNR was also relatively low (0.078 and 0.09 for deletions and tandem duplications, respectively). For our CNV pipeline, we found that FDR was very low (0 for deletions and duplications). However, FNR was comparatively higher at 0.16 for novel heterozygous deletions, 0.04 for novel homozygous deletions, and 0.36 for novel heterozygous duplications.

Pairwise genetic distances between SC lines

In order to ensure variation among genotypes or populations was not merely due to differences in genetic distances between lineages within or between each population, we calculated the pairwise genetic distances between each of the 9 genotypes (using the SC assemblies). Between populations, distances ranged from 0.004 to 0.005 and within populations, distances were between 0.001 and 0.002 (Table S13A in gray). We mapped reads from each of the 9 SC lines to the FASC assembly using BWA. We then used GATK *HaplotypeCaller* to assign genotypes for each site of each line separately. For each line, we required that all sites have a depth of coverage between 20 and 150. If the site was heterozygous, we additionally required i) the site be biallelic, ii) the minor allele frequency be ≥0.2, iii) the second largest phred-likelihood score be ≥30, and iv) at least one read possessing the alternate base on the forward and backward strand. We also required that a site be genotyped for all 9 SC lines. We then calculated pairwise genetic distances between each of the 9 SC lines (Table S13A). Pairwise-genetic distance was calculated by summing the number of sites between two samples that differed in genotype and dividing by the number of callable sites (Table S1). Pairs of sites that are homozygous for different alleles (homozygous differences) are weighted

twice as much (1) as pair of sites where one sample is homozygous and the other sample is heterozygous (0.5; heterozygous differences). Repeating this process when mapping to the GASC and IASC assembly did not qualitatively changed the results (Table S13B, S13C).

Supplemental Figures and Tables

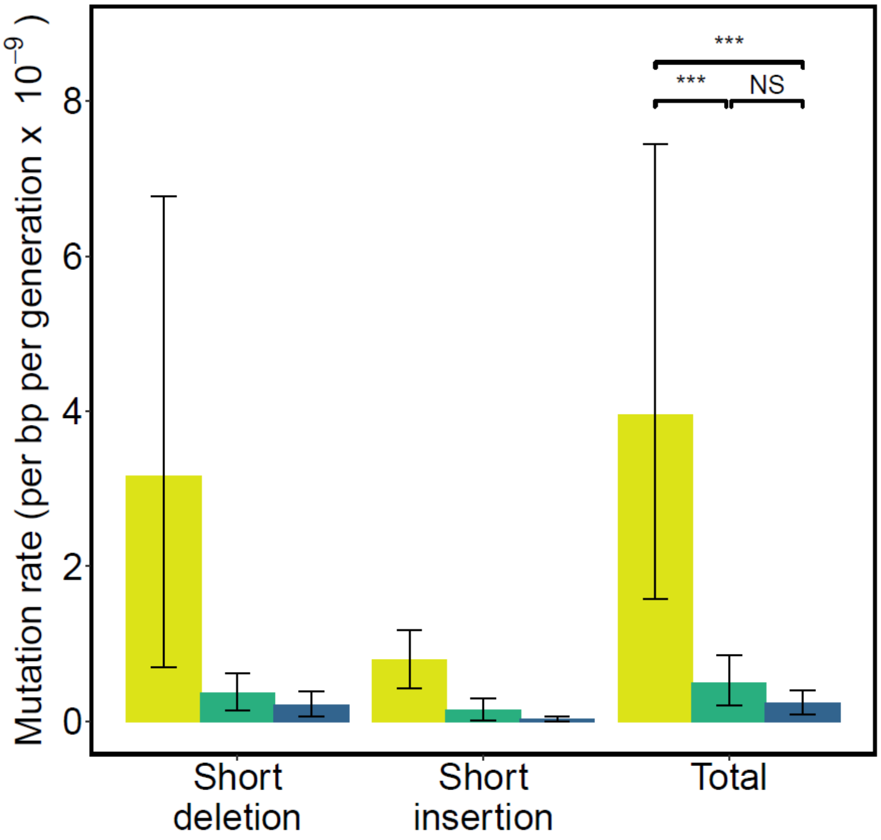

Figure S1. Mean mutation rates for short deletions (left), short insertions (middle), and total rates (right) for short indels (bars indicate 95% bootstrap confidence intervals) based on MA lines from three regions (Finland [yellow], Germany [green], and Israel [blue]). Results of post-hoc Tukey tests are indicated with brackets; 'NS' and '***' represents P-values > 0.05 and < 0.05, respectively.

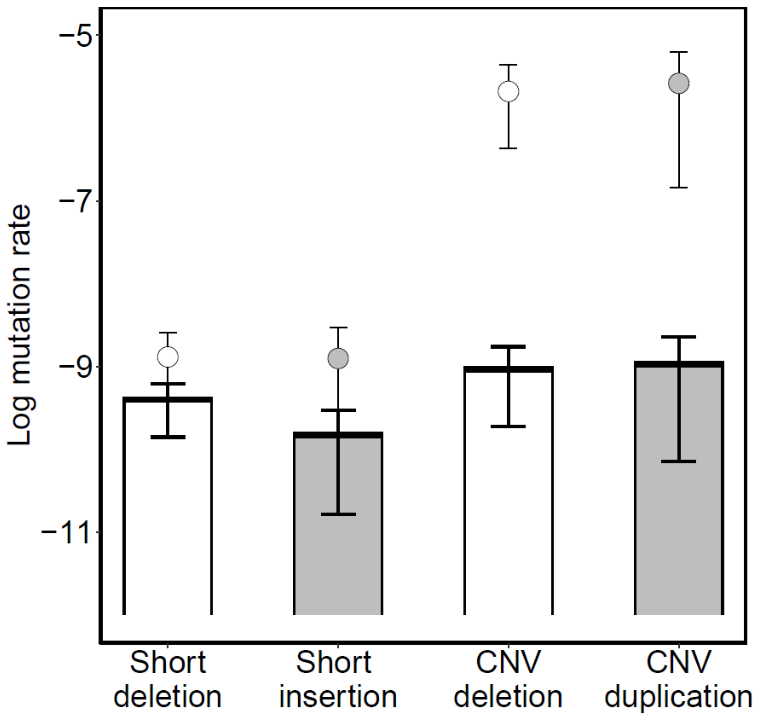

Figure S2. Mean mutation rates (+/- 95% bootstrap confidence intervals) for short indels and CNVs of German MA lines. Bar represents the count mutation rate in units of per bp per generation. Circles represent the length adjusted mutation rates in units of bp per bp per generation.

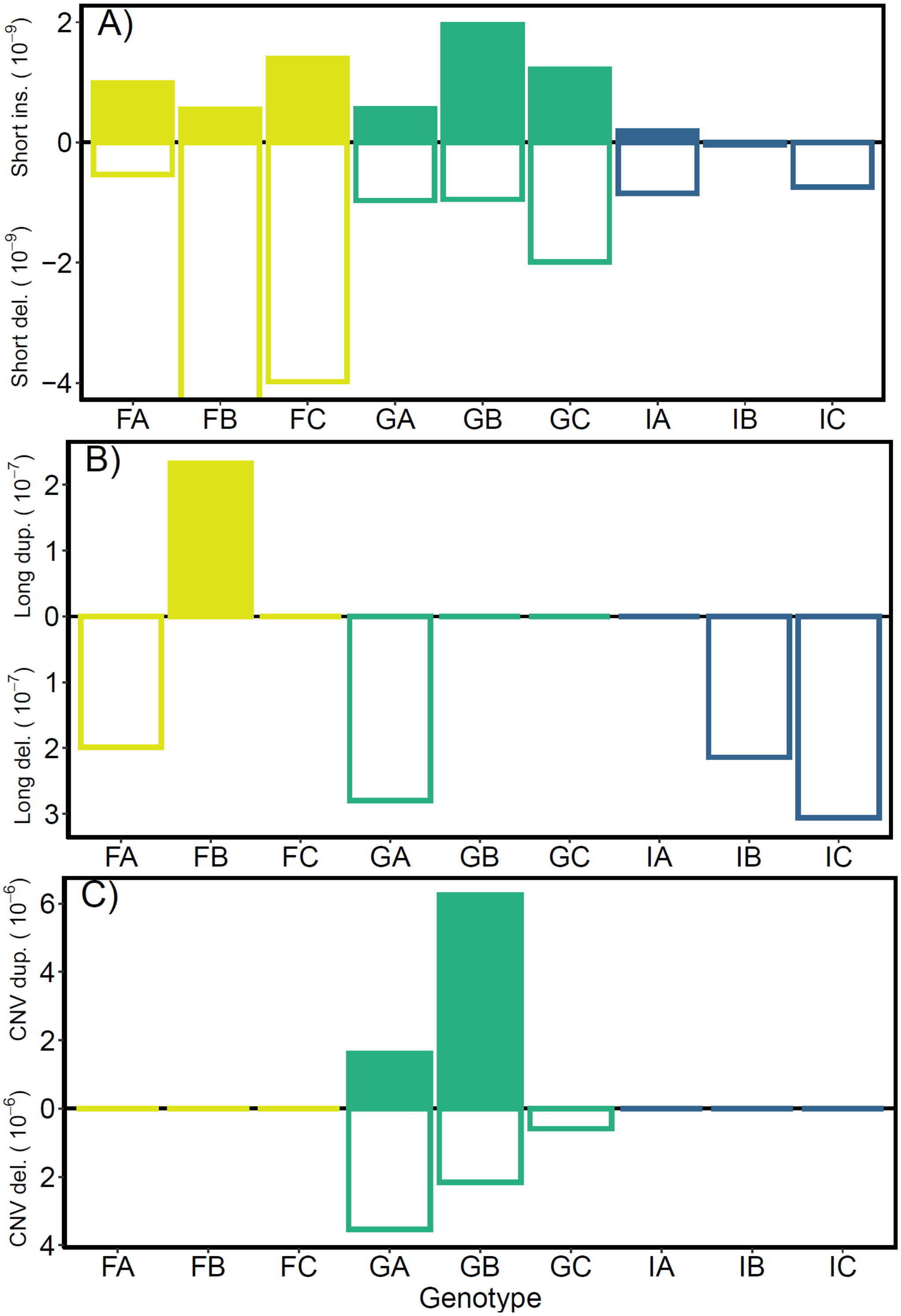

Figure S3. Length-adjusted mutation rates (bp per bp per generation) for six types of structural variants averaged across MA lines of each genotype. Mutations include (A) short indels (< 50 bp), (B) long deletion and tandem duplications (≥50 bp), and (C) CNV deletion and duplications (≥2000 bp). Yellow, green, and blue represent genotypes that originated from Finland, Germany, and Israel, respectively.

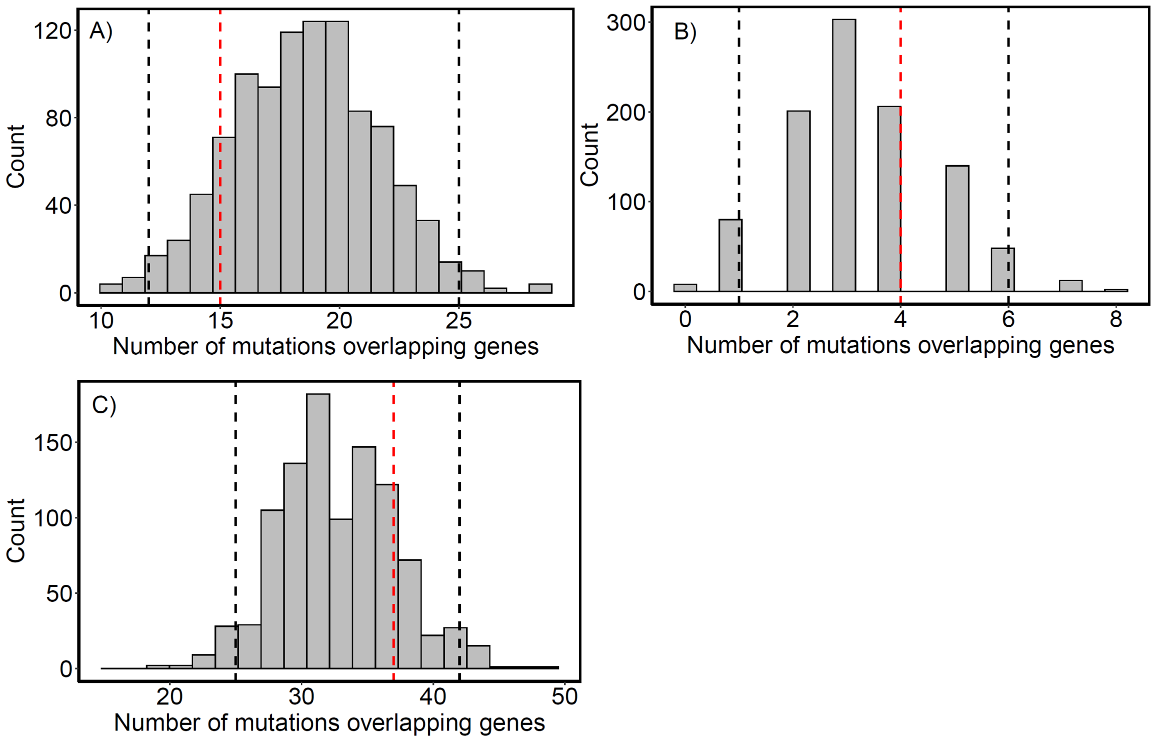

Figure S4. Distribution of the number of gene overlaps for simulated sets of mutations. (A) Short insertion and deletions. (B) Long deletions and tandem duplications. (C) CNV deletions and duplications. For each plot, the red dashed line indicates the number of overlaps in the observed mutation set. The black dash lines indicate the 2.5th and 97.5th percentiles for the number of overlaps in the simulated sets.

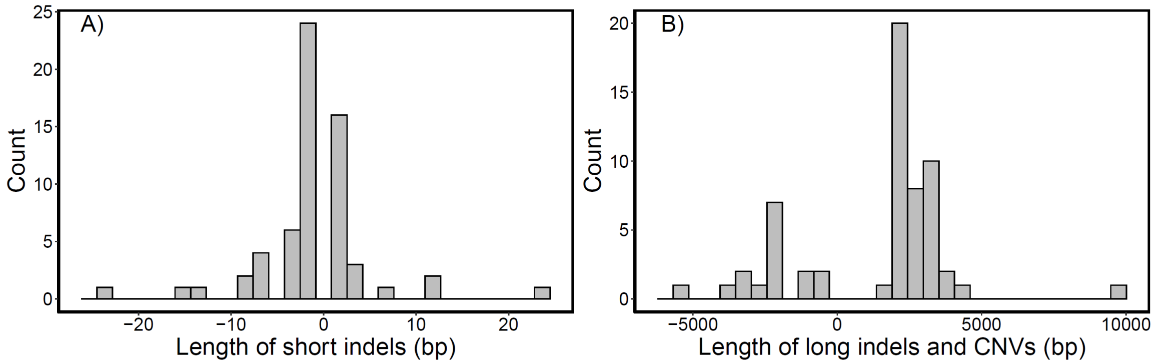

Figure S5. Distribution of lengths for (A) short indels (<50bp) and (B) long indels (>= 50bp) and CNVs identified in all MA lines of *D. magna*. Negative lengths indicate a deletion, while positive lengths indicate a duplication.

SUPPLEMENTAL REFERENCES

Altschul, S.F., Gish, W., Miller, W., Myers, E.W., Lipman, D.J., 1990. Basic local alignment search tool. J. Mol. Biol. 215, 403-410. https://doi.org/10.1016/S0022-2836(05)80360-2

Bankevich, A., Nurk, S., Antipov, D., Gurevich, A.A., Dvorkin, M., Kulikov, A.S., Lesin, V.M., Nikolenko, S.I., Pham, S., Prjibelski, A.D., Pyshkin, A.V., Sirotkin, A.V., Vyahhi, N., Tesler, G., Alekseyev, M.A., Pevzner, P.A., 2012. SPAdes: A new genome assembly algorithm and its applications to single-cell sequencing. J Comput Biol 19, 455–477. https://doi.org/10.1089/cmb.2012.0021

Bushnell, B., Rood, J., Singer, E., 2017. BBMerge – Accurate paired shotgun read merging via overlap. PLOS ONE 12, e0185056. https://doi.org/10.1371/journal.pone.0185056

Cantarel, B.L., Korf, I., Robb, S.M.C., Parra, G., Ross, E., Moore, B., Holt, C., Sanchez Alvarado, A., Yandell, M., 2008. MAKER: An easy-to-use annotation pipeline designed for emerging model organism genomes. Genome Research 18, 188–196. https://doi.org/10.1101/gr.6743907

Klambauer, G., Schwarzbauer, K., Mayr, A., Clevert, D.-A., Mitterecker, A., Bodenhofer, U., Hochreiter, S., 2012. cn.MOPS: mixture of Poissons for discovering copy number variations in next-generation sequencing data with a low false discovery rate. Nucleic Acids Research 40, e69–e69. https://doi.org/10.1093/nar/gks003

Klüttgen, B., Dülmer, U., Engels, M., Ratte, H.T., 1994. ADaM, an artificial freshwater for the culture of zooplankton. Water Research 28, 743–746. https://doi.org/10.1016/0043-1354(94)90157-0

Korf, I., 2004. Gene finding in novel genomes. BMC Bioinformatics 9.

Layer, R.M., Chiang, C., Quinlan, A.R., Hall, I.M., 2014. LUMPY: a probabilistic framework for structural variant discovery. Genome Biol 15, R84. https://doi.org/10.1186/gb-2014-15-6-r84

Li, H., Handsaker, B., Wysoker, A., Fennell, T., Ruan, J., Homer, N., Marth, G., Abecasis, G., Durbin, R., 1000 Genome Project Data Processing Subgroup, 2009. The sequence alignment/map format and SAMtools. Bioinformatics 25, 2078–2079. https://doi.org/10.1093/bioinformatics/btp352

McKenna, A., Hanna, M., Banks, E., Sivachenko, A., Cibulskis, K., Kernytsky, A., Garimella, K., Altshuler, D., Gabriel, S., Daly, M., DePristo, M.A., 2010. The Genome Analysis Toolkit: a MapReduce framework for analyzing next-generation DNA sequencing data. Genome Research 20, 1297–1303. https://doi.org/10.1101/gr.107524.110

Pryszcz, L.P., Gabaldón, T., 2016. Redundans: an assembly pipeline for highly heterozygous genomes. Nucleic Acids Res 44, e113–e113. https://doi.org/10.1093/nar/gkw294

R Core Team. 2018. R: A language and environment for statistical computing. R Foundation for Statistical Computing, Vienna, Austria. <https://www.R-project.org>.

Smit, A., Hubley, R., RepeatModeler Open-1.0. 2008-2015 <http://www.repeatmasker.org>.

Smit, A., Hubley, R., Green, P., RepeatMasker Open-4.0. 2013-2015 <http://www.repeatmasker.org>.

Stanke, M., Diekhans, M., Baertsch, R., Haussler, D., 2008. Using native and synthetically mapped cDNA alignments to improve de novo gene finding. Bioinformatics 24, 637–644. https://doi.org/10.1093/bioinformatics/btn013

Yampolsky, L.Y., Zeng, E., Lopez, J., Williams, P.J., Dick, K.B., Colbourne, J.K., Pfrender, M.E., 2014. Functional genomics of acclimation and adaptation in response to thermal stress in Daphnia. BMC Genomics 15, 859. https://doi.org/10.1186/1471-2164-15-859
